# On Consistency Test Method of Expert Opinion in Ecological Security Assessment

**DOI:** 10.3390/ijerph14091012

**Published:** 2017-09-04

**Authors:** Zaiwu Gong, Lihong Wang

**Affiliations:** Collaborative Innovation Center on Forecast and Evaluation of Meteorological Disasters, College of Economics and Management, Nanjing University of Information Science and Technology, Nanjing 210044, China; gongzilihong@163.com

**Keywords:** ecological security, sustainable development, group decision making, fuzzy preference relation, consensus measure

## Abstract

To reflect the initiative design and initiative of human security management and safety warning, ecological safety assessment is of great value. In the comprehensive evaluation of regional ecological security with the participation of experts, the expert’s individual judgment level, ability and the consistency of the expert’s overall opinion will have a very important influence on the evaluation result. This paper studies the consistency measure and consensus measure based on the multiplicative and additive consistency property of fuzzy preference relation (FPR). We firstly propose the optimization methods to obtain the optimal multiplicative consistent and additively consistent FPRs of individual and group judgments, respectively. Then, we put forward a consistency measure by computing the distance between the original individual judgment and the optimal individual estimation, along with a consensus measure by computing the distance between the original collective judgment and the optimal collective estimation. In the end, we make a case study on ecological security for five cities. Result shows that the optimal FPRs are helpful in measuring the consistency degree of individual judgment and the consensus degree of collective judgment.

## 1. Introduction

Ecological security refers to the overall level of ecosystem integrity and health, especially the minimum risk of survival and development, and the state of being not threatened. With the rapid growth of population and the development of social economy, the pressure of human activities on the environment is increasing, the contradiction between people and the earth is exacerbated, and the problem of excessive consumption of resources is becoming more and more serious. The threat of ecological damage and the environmental disasters caused by environmental degradation and ecological destruction to regional development, national security and social progress is increasing. Thus, the ecological security issue has been paid much attention on a global scale. The problem of ecological security has become a hot issue that needs to be solved urgently both in theory and practice [1,2,3,4,5,6,7].

The Yangtze River Delta urban agglomeration is the highest degree of urbanization, the most densely distributed towns and the highest level of economic development in China. Because the Yangtze River Delta urban agglomeration is located on the eastern coast of China and along the developed areas along the Yangtze River, the geographical advantages are prominent and the ecological status is very important. It plays an important role in maintaining the ecological balance of the middle and lower reaches of the Yangtze River and promoting the healthy development of the economy in the middle and lower reaches. In recent years, the economic development of the Yangtze River Delta region has shown a rapid growth trend, but, due to the degradation of the ecological environment and frequent ecological disasters, ecological security has become an important factor restricting the sustainable development of the Yangtze River Delta region. At present, the study on the ecological security of the Yangtze River Delta is still lacking. Therefore, it is urgent to carry out the comprehensive evaluation of the ecological security of the region, and then put forward the countermeasures to curb the deterioration of the ecological environment. Comprehensive evaluation of ecological security not only involves the natural environment, ecological and environmental disasters, environmental pollution, socio-economic and many other objective indicators, but also need to fully respect the subjective experience of experts and judgments. In the comprehensive evaluation of regional ecological security, the expert’s individual judgment level, ability and the consistency of the expert opinion (consensus level) have a very important influence on the evaluation result. This paper will study the consistency test method of expert opinion in ecological security assessment based on consensus decision theory.

In group decision making (GDM) analysis, it is often required to establish a procedure to aggregate multiple individual subjective preferences into an optimal consensus and then select the most favorable alternative. Usually, preference relations are widely used to express the subjective preference in group decision. In each preference relation, the decision maker (DM) is expected to provide a rational judgment or estimation; in a collective preference relation, the whole group of DMs is expected to reach a full agreement [8]. The first expectation leads to the consistency measure research for individual preference relations, and the second to the consensus measure research for collective preference relations. Obtaining a high level of consistency for an individual judgment and a high level of consensus between the DMs is a better choice. There are two main kinds of preference relations: multiplicative preference relation (reciprocal judgment matrix) [9,10] and fuzzy preference relation [11,12]. The original consistency framework of multiplicative preference relations was suggested by Saaty [9,10]: perfect consistency, acceptable consistency, consistency indexes, etc. To improve consistency, Saaty [9,10] compared each element of the given multiplicative preference relation with the ratio of weights. In [13], Lamata and Pelaez extended Saaty’s work. Considering the fact that the accuracy of the final ranking of the alternatives must satisfy the consistency ratio, many methods are proposed to properly adjust the multiplicative preference relation to an acceptable consistency index. Xu and Wei [14] proposed an algorithm to improve the consistency of the original multiplicative preference relation and prove that the algorithm is convergent. Dong et al. [15] extended the improved method to construct consensus models of a collective multiplicative preference relation under geometric mean prioritization method. Traditionally, this work is associated with the ratio of weights. Recently, Wang et al. [16] suggested a geometric mean method to obtain the perfectly consistent matrix from an indirect point of view. 

The consistency framework of FPR was constructed in, (e.g. [8,11,17,18]). There are two main consistency properties of the FPR. One is called multiplicative consistency, and the other is additive consistency. The central issue of these two kinds of consistencies includes three main aspects: (1) the priority of the FPR; (2) the estimation of the missing values in the FPR [18,19,20,21]; and (3) consistency measure and consensus measure of the FPRs [12,19,22,23,24,25,26,27,28,29,30,31,32,33,34,35,36,37]. The latter two aspects have been hotly discussed in recent years. For example, Xu [18] and Jiang et al. [21] developed different optimization methods to determine the priority vector of an incomplete FPR, and developed procedures for decision making based on incomplete FPR. Alonso et al. [24] presented an implemented web based consensus support system that is able to help the moderator in a consensus process where experts are allowed to provide their preferences using one of many types of incomplete preference relations. Chiclana et al. [31] presented a consensus model for GDM problems that proceeds from consistency to consensus, which is also a consensus framework for GDM based on additively consistent FPR. Dong et al. [32] extended Chiclana and coworkers’ work. In 2006, Ma et al. [21] established a method for repairing the inconsistency of FPRs from an indirect point of view. Xu and Cai [33] established several goal programming models and quadratic programming models based on minimizing deviation variables from the viewpoint of maximizing consensus.

In this paper, we derive an optimization method to obtain an optimal multiplicative consistent FPR and an optimal additively consistent FPR for individual and group judgments, respectively. We also propose a consistency measure by computing the distance between the original individual judgment and the optimal individual estimation, along with a consensus measure by computing the distance between the original collective judgment and the optimal collective estimation. 

The paper is organized as follows. In Section 2, we provide a brief description of the perfect consistency of two kinds of FPR. In Section 3, we propose two optimization models for constructing the multiplicative consistent FPR and the additively consistent FPR. In Section 4, we introduce a consistency measure for individual FPR. In Section 5, we firstly derive two methods for constructing the optimal collective multiplicative consistent FPR and the optimal collective additively consistent FPR, and then construct a consensus measure for collective FPR. In Section 6, we made a case study on ecological security; A short conclusion is given in Section 7.

## 2. Literature Review for the Consistency of Two Kinds of FPR

For simplicity, we denote N={1,2,…,n}, M={1,2,…,m}.

In decision making, pairwise comparisons are often used by the DMs to compare a set of decision alternatives with respect to a criterion. Different DMs may have different preferences. Usually, there are two main preferences used to quantify the comparative judgments. One is the reciprocal preference relation, which is initially proposed by Saaty [9,10]. For a set $X=\{x_{1},x_{2},\ldots ,x_{n}\}$ of alternatives, the preference information of pairwise comparisons with respect to a single criterion is represented numerically using a positive reciprocal matrix $P={\left(p_{ij}\right)}_{n\times n}$ on the scale of 1–9, where entry $p_{ij}$ estimates the preference degree or intensity of alternative $x_{i}$ over $x_{j}$, and satisfies $p_{ij}p_{ji}=1,p_{ij}>0$. Such a matrix is also called a multiplicative preference relation. Particularly, $p_{ij}=1$ indicates indifference between $x_{i}$ and $x_{j}$, $p_{ij}>1$ indicates $x_{i}$ is preferred to $x_{j}$, and $p_{ij}<1$ indicates $x_{j}$ is preferred to $x_{i}$. 

Another is the complementary preference relation, which is also called an FPR. It is firstly put forward by Orlovsky [38]. For alternatives $X=\{x_{1},x_{2},\ldots ,x_{n}\}$, the preference information of pairwise comparisons with respect to a single criterion is represented numerically using a complementary matrix $A={\left(a_{ij}\right)}_{n\times n}$ on the fuzzy scale of 0.1–0.9, where entry $a_{ij}$ estimates the preference degree or intensity of alternative $x_{i}$ over $x_{j}$, and satisfies $a_{ij}+a_{ji}=1,a_{ij}>0$. Such a matrix is called an FPR. Particularly, $a_{ij}=0.5$ indicates indifference between $x_{i}$ and $x_{j}$, $a_{ij}>0.5$ indicates $x_{i}$ is preferred to $x_{j}$, and $a_{ij}<0.5$ indicates $x_{j}$ is preferred to $x_{i}$. 

Let $P={\left(p_{ij}\right)}_{n\times n}$ be a multiplicative preference relation with $p_{ij}p_{ji}=1$ and $p_{ij}>0$ for i,j∈N. According to Saaty’s definition, $P={\left(p_{ij}\right)}_{n\times n}$ is perfectly consistent (also called multiplicative consistent) if, $p_{ij}=p_{ik}p_{kj}$ holds for k∈N. However, because: (1) the rating scale itself is discrete instead of continuous; (2) most decision-making may be complex; and (3) the DMs have to perform all the n(n−1)/2 required comparisons even when n is large, the DMs’ thinking may be inconsistent or illogical. Consequently, such consistency is hard to meet practically. Much work has been devoted to the construction of the consistent reciprocal preference relation. Recently, Wang et al. [16] propose an indirect judgments method to construct a perfectly consistent multiplicative preference relation $\hat{P}={\left({\hat{p}}_{ij}\right)}_{n\times n}$ by using geometric average ${\hat{p}}_{ij}=\prod_{k=1}^{n}\;{\left(p_{ik}p_{kj}\right)}^{\frac{1}{n}}$, i,j∈N. 

Let $A={\left(a_{ij}\right)}_{n\times n}$ be an FPR with $a_{ij}+a_{ji}=1,a_{ij}>0$ for i,j∈N. In 1984, Tanino [11] introduced $p_{ij}=a_{ij}/a_{ji}$ as a ratio of the preference intensity of $x_{i}$ to $x_{j}$. In this sense, $x_{i}$ is $a_{ij}/a_{ji}$ times as good as $x_{j}$. Thus, we have a new reciprocal preference relation $P={\left(p_{ij}\right)}_{n\times n}$ whose entries satisfy $p_{ij}p_{ji}=1,p_{ij}>0$ for i,j∈N. Thus, if $p_{ij}p_{jk}=p_{ik}$, that is, if

(1) $$\frac{a_{ij}}{a_{ji}}\frac{a_{jk}}{a_{kj}}=\frac{a_{ik}}{a_{ki}}$$

then $P={\left(p_{ij}\right)}_{n\times n}$ is also a multiplicative consistent preference relation. Tanino [11] defined Equation (1) as a multiplicative transitivity condition of the FPR $A={\left(a_{ij}\right)}_{n\times n}$. An FPR $A={\left(a_{ij}\right)}_{n\times n}$ satisfying Equation (1) is called a multiplicative consistent FPR [9,39], and much work has been done on the priority issues of the FPR [18,20,39,40,41]. Similar studies have been carried out combining consistency and preference relations, for example intuitionistic fuzzy preference relations [42,43]. Zhang [18], Tan and Gong [20], Wan et al. [39], Zhang [40], and Razmi [41] have done much work on the priority issues of the FPR. Tanino [11] also suggested another concept of transitivity for FPRs which is called an additive consistency. In particular, for an FPR $A={\left(a_{ij}\right)}_{n\times n}$, i,j,l∈N

(2) $$a_{ij}-0.5=a_{il}-0.5+a_{lj}-0.5$$

or equivalently,

(3) $$a_{ij}=a_{il}+a_{lj}-0.5$$

Usually, Equation (2) or Equation (3) is called the additively consistent condition of the FPR A. The additively consistent condition is very helpful in measuring the consistency or the consensus of the FPRs and in adding the missing information of the incomplete FPR, etc. For example, Herrera-Viedma et al. [8] gave a new characterization of consistency based on the additively consistent of FPRs, and showed that the new characterization of consistency can be readily checked by looking at the consistency in the DMs’ opinions. Alonso et al. [19,22,23,24] presented several consistency and consensus measures utilizing the additive consistency property to tackle missing information of incomplete preference relations. Ma et al. [21] also derived an indirect judgment method to construct an additively consistent preference relation $\hat{A}={\left({\hat{a}}_{ij}\right)}_{n\times n}$ by using arithmetic average ${\hat{a}}_{ij}=0.5+\frac{1}{n}(\sum_{l=1}^{n}\;a_{il}-a_{jl})$, i,j∈N. 

In the following section, based on the indirect methods of Wang et al. [16], Ma et al. [21], and Xu et al. [33], we derive two optimization models to construct the multiplicative consistent and additively consistent FPR. 

## 3. Optimization Methods for Constructing the Perfectly Consistent FPRs

### 3.1. A Logarithmic Least Squares Method for Constructing the Multiplicative Consistent FPR

Let $A={\left(a_{ij}\right)}_{n\times n}$ be an FPR whose entries satisfy $a_{ij}+a_{ji}=1,a_{ij}>0$, for all i,j∈N. If $A={\left(a_{ij}\right)}_{n\times n}$ is multiplicative consistent, then Equation (1) holds for all i,j,k∈N. Equation (1) is equivalent to

(4) $$log(\frac{a_{ij}}{a_{ji}})=log(\frac{a_{ik}a_{kj}}{a_{ki}a_{jk}})$$

However, $A={\left(a_{ij}\right)}_{n\times n}$ may be not multiplicative consistent, which denotes that $log(\frac{a_{ij}}{a_{ji}})\neq log(\frac{a_{ik}a_{kj}}{a_{ki}a_{jk}})$. Let $l_{ij}=|log(\frac{a_{ij}}{a_{ji}})-log(\frac{a_{ik}a_{kj}}{a_{ki}a_{jk}})|$ denote the logarithmic distance between $\frac{a_{ij}}{a_{ji}}$ and $\frac{a_{ik}a_{kj}}{a_{ki}a_{jk}}$. Obviously, the smaller the squared distance $l_{ij}$ is, the better consistency of the FPR $A={\left(a_{ij}\right)}_{n\times n}$. This also denotes the better estimation by the DM. An ideal estimation ${\hat{a}}_{ij}$ should be the optimal solution such that the sum of the squared distances between $log({\hat{a}}_{ij}/{\hat{a}}_{ji})$ and $log(\frac{a_{ik}a_{kj}}{a_{ki}a_{jk}})$, i,j,k∈N, is the minimum. Thus, we have the following optimization model:

(5) $$min\;l_{ij}^{2}=\sum_{k=1}^{n}\;{\left[log({\hat{a}}_{ij}/{\hat{a}}_{ji})-log(a_{ik}a_{kj}/a_{ki}a_{jk})\right]}^{2}\;s.t.\;0<{\hat{a}}_{ij}<1$$

Model (5) can be easily solved, leading to the unique solution

(6) $$log({\hat{a}}_{ij}/{\hat{a}}_{ji})=\frac{1}{n}\sum_{k=1}^{n}\;log(a_{ik}a_{kj}/a_{ki}a_{jk})$$

That is,

(7) $${\hat{a}}_{ij}/{\hat{a}}_{ji}=\prod_{k=1}^{n}\;{\left(a_{ik}a_{kj}/a_{ki}a_{jk}\right)}^{\frac{1}{n}}$$

If ${\hat{a}}_{ij}+{\hat{a}}_{ji}=1$, we then have

(8) $${\hat{a}}_{ij}=\frac{\prod_{k=1}^{n}\;{\left(a_{ik}a_{kj}/a_{ki}a_{jk}\right)}^{\frac{1}{n}}}{1+\prod_{k=1}^{n}\;{\left(a_{ik}a_{kj}/a_{ki}a_{jk}\right)}^{\frac{1}{n}}}$$

for all i,j∈N.

**Theorem 1.**  *If* ${\hat{a}}_{ij}+{\hat{a}}_{ji}=1$ *for all* i,j∈N*, then the optimization Model (5) has a unique solution* ${\hat{a}}_{ij}=\prod_{k=1}^{n}\;{\left(a_{ik}a_{kj}/a_{ki}a_{jk}\right)}^{\frac{1}{n}}/(1+\prod_{k=1}^{n}\;{\left(a_{ik}a_{kj}/a_{ki}a_{jk}\right)}^{\frac{1}{n}})$*.*

Let us now construct a new matrix $\hat{A}={\left({\hat{a}}_{ij}\right)}_{n\times n}$ such that ${\hat{a}}_{ij}=\frac{\prod_{k=1}^{n}\;{\left(a_{ik}a_{kj}/a_{ki}a_{jk}\right)}^{\frac{1}{n}}}{1+\prod_{k=1}^{n}\;{\left(a_{ik}a_{kj}/a_{ki}a_{jk}\right)}^{\frac{1}{n}}}$, i,j∈N. In the following, we prove that matrix $\hat{A}={\left({\hat{a}}_{ij}\right)}_{n\times n}$ is an FPR and satisfies the multiplicative consistency condition (1).

(I). For all i∈N, we have
  $${\hat{a}}_{ii}=0.5$$
(II). For all i,j∈N, we have
  $${\hat{a}}_{ij}+{\hat{a}}_{ji}=1$$
(III). For all i,j∈N, we have
  $$0<{\hat{a}}_{ij}<1$$
(IV). For all i,j,k∈N, we have
  $${\hat{a}}_{ij}/{\hat{a}}_{ji}=\prod_{l=1}^{n}\;{\left(a_{il}a_{lj}/a_{li}a_{jl}\right)}^{\frac{1}{n}},\;{\hat{a}}_{ki}/{\hat{a}}_{ik}=\prod_{l=1}^{n}\;{\left(a_{kl}a_{li}/a_{lk}a_{il}\right)}^{\frac{1}{n}},$$
  and
  $${\hat{a}}_{jk}/{\hat{a}}_{kj}=\prod_{l=1}^{n}\;{\left(a_{jl}a_{lk}/a_{lj}a_{kl}\right)}^{\frac{1}{n}}.$$

Thus, we have

(9) $${\hat{a}}_{ij}{\hat{a}}_{ki}{\hat{a}}_{jk}/{\hat{a}}_{ji}{\hat{a}}_{ik}{\hat{a}}_{kj}=\prod_{l=1}^{n}\;{\left(a_{il}a_{lj}a_{kl}a_{li}a_{jl}a_{lk}/a_{li}a_{jl}a_{lk}a_{il}a_{lj}a_{kl}\right)}^{\frac{1}{n}}=1$$

which denotes that the condition $\frac{{\hat{a}}_{ij}}{{\hat{a}}_{ji}}\frac{{\hat{a}}_{jk}}{{\hat{a}}_{kj}}=\frac{{\hat{a}}_{ik}}{{\hat{a}}_{ki}}$ holds for all i,j,k∈N. 

Consequently, the matrix $\hat{A}={\left({\hat{a}}_{ij}\right)}_{n\times n}$ is an FPR which satisfies multiplicative consistent condition following from (I), (III) and (III) and (IV).

**Theorem 2.**  *The matrix* $\hat{A}={\left({\hat{a}}_{ij}\right)}_{n\times n}$ *is an FPR which satisfies the multiplicative consistency, where* ${\hat{a}}_{ij}=\frac{\prod_{k=1}^{n}\;{\left(a_{ik}a_{kj}/a_{ki}a_{jk}\right)}^{\frac{1}{n}}}{1+\prod_{k=1}^{n}\;{\left(a_{ik}a_{kj}/a_{ki}a_{jk}\right)}^{\frac{1}{n}}}$*,* i,j∈N*.*

We call the multiplicative consistent FPR $\hat{A}={\left({\hat{a}}_{ij}\right)}_{n\times n}$ satisfying ${\hat{a}}_{ij}=\frac{\prod_{k=1}^{n}\;{\left(a_{ik}a_{kj}/a_{ki}a_{jk}\right)}^{\frac{1}{n}}}{1+\prod_{k=1}^{n}\;{\left(a_{ik}a_{kj}/a_{ki}a_{jk}\right)}^{\frac{1}{n}}}$, i,j∈N, the optimal estimation matrix (or the optimal multiplicative consistent FPR) of $A={\left(a_{ij}\right)}_{n\times n}$. In fact, $\hat{A}$ can be regarded as an objective estimation of matrix A.

### 3.2. The Optimal Additively Consistent FPR

Let $A={\left(a_{ij}\right)}_{n\times n}$ be an FPR whose entries satisfy $a_{ij}+a_{ji}=1,a_{ij}>0$, for all i,j∈N. If $A={\left(a_{ij}\right)}_{n\times n}$ is additively consistent, then Equation (3) holds for all i,j,k∈N. However, $A={\left(a_{ij}\right)}_{n\times n}$ may be not additively consistent, which denotes that $a_{ij}\neq a_{il}+a_{lj}-0.5$. Let $d_{ij}=|a_{ij}-(a_{il}+a_{lj}-0.5)|$ denote the distance between $a_{ij}$ and $a_{il}+a_{lj}-0.5$. It is obvious that the smaller the squared distance $d_{ij}$ is, the better consistency of the estimation by the DM. Suppose ${\hat{a}}_{ij}$ is an optimal estimation such that the sum of the squared distance between ${\hat{a}}_{ij}$ and $a_{il}+a_{lj}-0.5$, i,j∈N is the minimum. Thus we have the following optimization model

(10) $$min\;d_{ij}^{2}=\sum_{i=1}^{n}\;{\left({\hat{a}}_{ij}-(a_{il}+a_{lj}-0.5)\right)}^{2}\;s.t.\;0<{\hat{a}}_{ij}<1$$

We can readily see that

(11) $${\hat{a}}_{ij}=\frac{1}{n}\sum_{l=1}^{n}\;(a_{il}+a_{lj}-0.5)$$

is the unique solution to Model (10).

**Theorem 3.**  *The optimal Model (10) has a unique solution* ${\hat{a}}_{ij}=\frac{1}{n}\sum_{l=1}^{n}(a_{il}+a_{lj}-0.5)$*.*

Let us construct a new matrix $\hat{A}={\left({\hat{a}}_{ij}\right)}_{n\times n}$ such that ${\hat{a}}_{ij}=\frac{1}{n}\sum_{l=1}^{n}\;(a_{il}+a_{lj}-0.5)$, i,j∈N. In the following, it is proven that matrix $\hat{A}={\left({\hat{a}}_{ij}\right)}_{n\times n}$ is an FPR and satisfies the additive consistency condition.

(I). For all i∈N, we have
  $${\hat{a}}_{ii}=\frac{1}{n}\sum_{l=1}^{n}\;(a_{il}+a_{li}-0.5)=0.5.$$
(II). For all i,j∈N,
  $${\hat{a}}_{ij}=\frac{1}{n}\sum_{l=1}^{n}\;(a_{il}+a_{lj}-0.5),$$
  and
  $${\hat{a}}_{ji}=\frac{1}{n}\sum_{l=1}^{n}\;(a_{jl}+a_{li}-0.5).$$

We can readily see that

(12) $${\hat{a}}_{ij}+{\hat{a}}_{ji}=\frac{1}{n}\sum_{l=1}^{n}\;(a_{il}+a_{li}+a_{lj}+a_{jl})-1=1$$

(III). For all i,j∈N,
  $${\hat{a}}_{ik}=\frac{1}{n}\sum_{l=1}^{n}\;(a_{il}+a_{lk}-0.5),$$
  and
  $${\hat{a}}_{kj}=\frac{1}{n}\sum_{l=1}^{n}\;(a_{kl}+a_{lj}-0.5).$$

Thus,

(13) $${\hat{a}}_{ik}+{\hat{a}}_{kj}=\frac{1}{n}\sum_{l=1}^{n}\;(a_{il}+a_{lj}+a_{lk}+a_{kl})-1$$

(14) $$=\frac{1}{n}\sum_{l=1}^{n}\;(a_{il}+a_{lj})$$

Meanwhile, we have

(15) $${\hat{a}}_{ij}=\frac{1}{n}\sum_{l=1}^{n}\;(a_{il}+a_{lj}-0.5)\;=\frac{1}{n}\sum_{l=1}^{n}\;(a_{il}+a_{lj})-0.5\;={\hat{a}}_{ik}+{\hat{a}}_{kj}-0.5$$

Consequently, if $0<{\hat{a}}_{ij}<1$, then the matrix $\hat{A}={\left({\hat{a}}_{ij}\right)}_{n\times n}$ is an FPR and satisfies consistent condition following from (I), (III) and (III).

**Theorem 4.**  *The matrix* $\hat{A}={\left({\hat{a}}_{ij}\right)}_{n\times n}$ *is an FPR and possesses the additive consistency if* $0<{\hat{a}}_{ij}<1$*, where* ${\hat{a}}_{ij}=\frac{1}{n}\sum_{l=1}^{n}\;(a_{il}+a_{lj}-0.5)$*,* i,j∈N*.*

We also call the additively consistent FPR $\hat{A}={\left({\hat{a}}_{ij}\right)}_{n\times n}$ satisfying ${\hat{a}}_{ij}=\frac{1}{n}\sum_{l=1}^{n}\;(a_{il}+a_{lj}-0.5)$, i,j∈N, the optimal estimation matrix (or the optimal additively consistent FPR) of $A={\left(a_{ij}\right)}_{n\times n}$. In fact, $\hat{A}$ can be regarded as an objective estimation of matrix A.

## 4. The Consistency Measure of the Individual FPRs

Let $A={\left(a_{ij}\right)}_{n\times n}$ be an FPR, and $\hat{A}={\left({\hat{a}}_{ij}\right)}_{n\times n}$ the corresponding optimal estimation matrix calculated using Equation (8) or Equation (11). If $A={\left(a_{ij}\right)}_{n\times n}$ is not consistent, then there exists at least one pair i,j∈N such that ${\hat{a}}_{ij}\neq a_{ij}$. The smaller deviation between ${\hat{a}}_{ij}$ and $a_{ij}$ is, the better consistency of the estimation. This also denotes that the bigger deviation between ${\hat{a}}_{ij}$ and $a_{ij}$ is, the higher inconsistency of the estimation. Let $\delta_{ij}=|{\hat{a}}_{ij}-a_{ij}|$, for all i,j∈N. In the following, three consistent measures between the original FPR and its optimal estimation matrix are proposed. 

(I). Consistency degree associated with a pair of alternatives $x_{i}$ and $x_{j}$.

**Definition 1.**  *We call* $c_{ij}=1-\delta_{ij}$ *the consistency degree associated with the pair of alternative* $x_{i}$ *and* $x_{j}$*, and* $CM={\left(c_{ij}\right)}_{n\times n}$ *the consistency degree matrix associated with all the pair of alternatives* $x_{i}$ *and* $x_{j}$*,* i,j∈N*.*

In fact, $\delta_{ij}=|{\hat{a}}_{ij}-a_{ij}|$ is an inconsistency degree measure between $a_{ij}$ and its optimal estimation ${\hat{a}}_{ij}$, for all i,j∈N. The smaller $\delta_{ij}$ (or the bigger $1-\delta_{ij}$) is, the higher consistency of the estimation associated with $a_{ij}$. 

(II). Consistency degree associated with an alternative $x_{i}$.

**Definition 2.**  *We call* $c_{i}=\sum_{j=1,j\neq i}^{n}(1-\delta_{ij})/(n-1)$ *the consistency degree associated with the alternative* $x_{i}$*, and* $CV={\left(c_{1}c_{2}\ldots \;c_{n}\right)}^{T}$ *the consistency degree vector associated with all the alternatives.*

The consistency degree $c_{i}$ is actually an index for measuring the consistency degree between the original estimation and its optimal estimation for the alternative $x_{i}$. In other words, the bigger the value of $c_{i}$ is, the higher consistency of the estimation associated with the alternative $x_{i}$, i∈N. 

(III). Consistency degree of the FPR $A={\left(a_{ij}\right)}_{n\times n}$.

**Definition 3.**  *Let* $A={\left(a_{ij}\right)}_{n\times n}$ *be an FPR, and* $\hat{A}={\left({\hat{a}}_{ij}\right)}_{n\times n}$ *the corresponding optimal estimation matrix calculated using Equation (8) or Equation (11). We call* $C=\sum_{i=1}^{n}\;c_{i}/n$ *the consistency degree of the FPR* $A={\left(a_{ij}\right)}_{n\times n}$*.*

The bigger the value $c_{i}$ is, the higher consistency of the FPR $A={\left(a_{ij}\right)}_{n\times n}$. This consistent measure is similar to that of Chiclana et al. [31].

**Example 1.**  *The FPR* A *presented by Chiclana* [31] *is given below:*

$$A=\left(\begin{array}{cccc} 0.5 & 0.7 & 0.9 & 0.5\\ 0.3 & 0.5 & 0.6 & 0.7\\ 0.1 & 0.4 & 0.5 & 0.8\\ 0.5 & 0.3 & 0.2 & 0.5 \end{array}\right)$$

From Equation (11), the optimal estimation matrix of A is

$$\hat{A}=\left(\begin{array}{cccc} 0.5000 & 0.6250 & 0.7000 & 0.7750\\ 0.3750 & 0.5000 & 0.5750 & 0.6500\\ 0.3000 & 0.4250 & 0.5000 & 0.5750\\ 0.2250 & 0.3500 & 0.4250 & 0.5000 \end{array}\right)$$

The consistency measures of A as proposed in this paper and proposed by Chiclana are listed in Table 1. 

Here, we have two different consistency measures. The first consistency measure (our measure) is based on the optimal estimation matrix. That is, we regard this matrix as an objective matrix. The consistency degree of the FPR is based on the differences between the collective FPR (aggregated by arithmetic average of individual FPRs) and its optimal estimation FPR. In this sense, the first consistency measure is constructed from the point of view of collective divergence. The second consistency (Chiclana’s measure [31]) is based on the differences among all the individual FPRs. In this sense, the second consistency measure is constructed from the point of view of individual divergence. Consequently, the numerical values of the first consistency measure are greater than that of the second consistency measure, as shown in Table 1.

## 5. The Consensus Measure of Collective FPR

In group decision making, the collective preference relation is very helpful in selecting the best alternative. Usually, when aggregating the individual preference relations to a collective preference relation, the consensus measure is desirable. The consensus index is a useful tool for measuring the degree of consensus between the individual preference estimations and collective preference estimation. Dong et al. [15] defined the geometric cardinal consensus index and the geometric ordinal consensus index for AHP group decision making using RGMM (row geometric mean method). Chiclana et al. [31] measured the consensus degree by calculating the similarity between the preferences of each DMs in the group and the collective preferences. They also suggested that consistency needs to be checked before the application of the consensus process. In this section, using the consistent FPR derived by Section 3, we put forward consensus indexes to measure the consensus degree between individual and collective FPRs. 

### 5.1. Construction of Two Kinds of Collective Consistent FPRs

In group decision making, the weighted geometric average and weighted arithmetic average methods are often used to aggregate different individual preferences into a collective preference. In this section, we show by two theorems that the optimal estimation matrix for the aggregated FPR is equivalent to the aggregated optimal estimation matrix for different individual FPRs. 

Let $A_{s}={\left(a_{ijs}\right)}_{n\times n}$ be an FPR presented by DM $d_{s}$, and the corresponding weight of DM $d_{s}$ be $\omega_{s}$, where $\sum_{s=1}^{m}\;\omega_{s}=1$, s∈M. We introduce an auxiliary matrix $B_{s}={\left(b_{ijs}\right)}_{n\times n}$, where $b_{ijs}=\frac{a_{ijs}}{a_{jis}}$, s∈M. In fact, $B_{s}={\left(b_{ijs}\right)}_{n\times n}$ is a multiplicative preference relation.

**Lemma 1.**  $B_{s}={\left(b_{ijs}\right)}_{n\times n}$ *is a perfectly consistent preference relation if* $A_{s}={\left(a_{ijs}\right)}_{n\times n}$*,* s∈M*, is multiplicative consistent.*

Let $b_{ij}=\prod_{s=1}^{m}\;{\left(\frac{a_{ijs}}{a_{jis}}\right)}^{\omega_{s}}$, then we call $B={\left(b_{ij}\right)}_{n\times n}$ the weighted geometric average combination of $B_{s}={\left(b_{ijs}\right)}_{n\times n}$.

**Lemma 2.**  $B={\left(b_{ij}\right)}_{n\times n}$ *is perfectly consistent if* $A_{s}={\left(a_{ijs}\right)}_{n\times n}$*,* s∈M*, is multiplicative consistent.*

Lemmas 1 and 2 are obvious, the proof is omitted. 

Let us consider an FPR $A={\left(a_{ij}\right)}_{n\times n}$ whose entries satisfy $a_{ij}=\frac{b_{ij}}{1+b_{ij}}$, i,j∈N. Then $B={\left(b_{ij}\right)}_{n\times n}$ is an auxiliary matrix of $A={\left(a_{ij}\right)}_{n\times n}$ for the reason that $b_{ij}=\frac{a_{ij}}{a_{ji}}$, i,j∈N. 

For $a_{ij}=\frac{b_{ij}}{1+b_{ij}}$, we have

(16) $$a_{ij}=\frac{\prod_{s=1}^{m}\;{\left(\frac{a_{ijs}}{a_{jis}}\right)}^{\omega_{s}}}{1+\prod_{s=1}^{m}\;{\left(\frac{a_{ijs}}{a_{jis}}\right)}^{\omega_{s}}}$$

(17) $$=\frac{\prod_{s=1}^{m}\;{\left(a_{ijs}\right)}^{\omega_{s}}}{\prod_{s=1}^{m}\;{\left(a_{ijs}\right)}^{\omega_{s}}+\prod_{s=1}^{m}\;{\left(a_{jis}\right)}^{\omega_{s}}}$$

Equation (17) is equivalent to

(18) $$\frac{a_{ij}}{a_{ji}}=\prod_{s=1}^{m}\;{\left(\frac{a_{ijs}}{a_{jis}}\right)}^{\omega_{s}}$$

and $a_{ij}+a_{ji}=1,0<a_{ij}<1$. 

Consequently, we define $A={\left(a_{ij}\right)}_{n\times n}$ as a weighted (particular) geometric average combination of $A_{s}={\left(a_{ijs}\right)}_{n\times n}$, s=1,2,…,m, if Equation (17) holds for all i,j∈N. It is ready to prove that if $A_{s}={\left(a_{ijs}\right)}_{n\times n}$ is an FPR, s=1,2,…,m, then $A={\left(a_{ij}\right)}_{n\times n}$ is an FPR.

(I). Suppose that $A_{s}={\left(a_{ijs}\right)}_{n\times n}$ is multiplicative consistent, for all s=1,2,…,m. By Lemma 2, $B={\left(b_{ij}\right)}_{n\times n}$ is perfectly consistent. Then, $b_{ij}b_{jk}=b_{ik}$ holds for all i,j,k∈N, which is equivalent to that $\frac{a_{ij}a_{jk}a_{ki}}{a_{ji}a_{kj}a_{ik}}=1$ holds for all i,j,k∈N. This denotes that $A={\left(a_{ij}\right)}_{n\times n}$ is a multiplicative consistent FPR. Thus, we have the following theorem.

**Theorem 5.**  *Let* $A_{s}={\left(a_{ijs}\right)}_{n\times n}$ *be an FPR,* s=1,2,…,m*, then the weighted geometric average combination* $A={\left(a_{ij}\right)}_{n\times n}$ *is also an FPR, where* $a_{ij}$ *satisfies Equation (17),* i,j∈N*. Moreover, if all the individual FPRs* $A_{s}={\left(a_{ijs}\right)}_{n\times n}$*,* s=1,2,…,m*, are multiplicative consistent, then the weighted geometric average combination* $A={\left(a_{ij}\right)}_{n\times n}$ *is also multiplicative consistent, where* $a_{ij}$ *satisfies Equation (17),* i,j∈N*.*

(II). Suppose there is at least one $A_{s}={\left(a_{ijs}\right)}_{n\times n}$ that is not multiplicative consistent, for some s∈M. Then, the weighted geometric average combination $A={\left(a_{ij}\right)}_{n\times n}$ may not be multiplicative consistent either, where Equation (17) holds for all i,j∈N. In many practical situations, it is hard for each DM to directly present his/her multiplicative consistent fuzzy preference; and it is also hard to directly obtain a collective multiplicative consistent FPR. Consequently, finding an indirectly algorithm to reconstruct multiplicative consistent FPRs is a practically better choice.

On the one hand, by Equation (7), the optimal estimation matrix $\hat{A}={\left({\hat{a}}_{ij}\right)}_{n\times s}$ of $A={\left(a_{ij}\right)}_{n\times n}$ satisfies

(19) $$\frac{{\hat{a}}_{ij}}{{\hat{a}}_{ji}}=\prod_{k=1}^{n}\;{\left(a_{ik}a_{kj}/a_{ki}a_{jk}\right)}^{\frac{1}{n}}\;=\prod_{k=1}^{n}\;\left(\prod_{s=1}^{m}\;\left(\frac{a_{iks}}{a_{kis}}\right){}^{\omega_{s}}\prod_{s=1}^{m}\;\left(\frac{a_{kjs}}{a_{jks}}\right){}^{\omega_{s}}\right){}^{\frac{1}{n}}\;=\prod_{k=1}^{n}\;\prod_{s=1}^{m}\;\left(\frac{a_{iks}a_{kjs}}{a_{kis}a_{jks}}\right){}^{\frac{\omega_{s}}{n}}$$

By Theorem 2, $\hat{A}={\left({\hat{a}}_{ij}\right)}_{n\times s}$ is multiplicative consistent. 

On the other hand, the optimal estimation matrix $A_{s}={\left({\hat{a}}_{ijs}\right)}_{n\times s}$ of $A_{s}={\left(a_{ijs}\right)}_{n\times n}$ satisfies

(20) $$\frac{{\hat{a}}_{ijs}}{{\hat{a}}_{jis}}=\prod_{k=1}^{n}\;\left(\frac{a_{iks}a_{kjs}}{a_{kis}a_{jks}}\right){}^{\frac{1}{n}}$$

Suppose that the weighted geometric average combination of $A_{s}={\left({\hat{a}}_{ijs}\right)}_{n\times s}$, s∈M, is $\hat{A^{\prime }}={\left({{\hat{a}}^{\prime }}_{ij}\right)}_{n\times n}$, where $\frac{{{\hat{a}}^{\prime }}_{ij}}{{{\hat{a}}^{\prime }}_{ji}}=\prod_{s=1}^{m}\;{\left(\frac{{\hat{a}}_{ijs}}{{\hat{a}}_{jis}}\right)}^{a_{s}}$. Then, we have

(21) $$\frac{{{\hat{a}}^{\prime }}_{ij}}{{{\hat{a}}^{\prime }}_{ji}}=\prod_{s=1}^{m}\left(\frac{{\hat{a}}_{ijs}}{{\hat{a}}_{jis}}\right){}^{\omega_{s}}\;=\prod_{s=1}^{m}\left(\prod_{k=1}^{n}\;\left(\frac{a_{iks}a_{kjs}}{a_{kis}a_{jks}}\right){}^{\frac{1}{n}}\right){}^{\omega_{s}}\;=\prod_{s=1}^{n}\;\prod_{k=1}^{m}\;\left(\frac{a_{iks}a_{kjs}}{a_{kis}a_{jks}}\right){}^{\frac{\omega_{s}}{n}}\;=\prod_{k=1}^{n}\;\prod_{s=1}^{m}\;\left(\frac{a_{iks}a_{kjs}}{a_{kis}a_{jks}}\right){}^{\frac{\omega_{s}}{n}}$$

Combining Equation (19) with Equation (21), we have that $\hat{A}=\hat{A^{\prime }}$. This means that ${\hat{A}}^{\prime }={\left({{\hat{a}}^{\prime }}_{ij}\right)}_{n\times n}$ is also multiplicative consistent. Now, the following Theorem 6 follows from the above process of reasoning.

**Theorem 6.**  The optimal estimation matrix of the weighted geometric average combination of individual FPRs is equivalent to the weighted geometric average combination of the optimal estimation matrix of the individual FPRs.

Let $A_{s}={\left(a_{ijs}\right)}_{n\times n}$ be an FPR presented by DM $d_{s}$, and the corresponding weight of the DM $d_{s}$ be $\omega_{s}$, where $\sum_{i=1}^{m}\;\omega_{s}=1$, s∈M. Then, the weighted arithmetic average combination $A={\left(a_{ij}\right)}_{n\times n}$ of $A_{s}={\left(a_{ijs}\right)}_{n\times n}$, s∈M, is also an FPR, where $a_{ij}=\sum_{i=1}^{m}\;\omega_{s}a_{ijs}$. If $A_{s}={\left(a_{ijs}\right)}_{n\times n}$ is additively consistent, then we can readily prove that $A={\left(a_{ij}\right)}_{n\times n}$ is also additively consistent. Thus we have the following theorem.

**Theorem 7.**  $A={\left(a_{ij}\right)}_{n\times n}$ *is an FPR if* $A_{s}={\left(a_{ijs}\right)}_{n\times n}$ *is an FPR, for all* s=1,2,…,m*;* $A={\left(a_{ij}\right)}_{n\times n}$ *is additively consistent if* $A_{s}={\left(a_{ijs}\right)}_{n\times n}$ *is additively consistent, for all* s=1,2,…,m*, where* $a_{ij}=\sum_{i=1}^{m}\;\omega_{s}a_{ijs}$*,* i,j∈N*.*

According to Equation (11), the optimal estimation matrix for $A={\left(a_{ij}\right)}_{n\times n}$ is $\hat{A}={\left({\hat{a}}_{ij}\right)}_{n\times n}$, where

(22) $${\hat{a}}_{ij}=\frac{1}{n}\sum_{l=1}^{n}\;(a_{il}+a_{lj}-0.5)$$

The optimal estimation matrix for $A_{s}={\left(a_{ijs}\right)}_{n\times n}$ is $A_{s}={\left({\hat{a}}_{ijs}\right)}_{n\times n}$, where

(23) $${\hat{a}}_{ijs}=\frac{1}{n}\sum_{l=1}^{n}\;(a_{ils}+a_{ljs}-0.5)$$

Then, the arithmetic average of ${\hat{A}}_{s}={\left({\hat{a}}_{ijs}\right)}_{n\times n}$, s=1,2,…,m is $\hat{\bar{A}}={\left({\hat{\bar{a}}}_{ij}\right)}_{n\times n}$, where

(24) $${\hat{\bar{a}}}_{ij}=\sum_{s=1}^{m}\;\omega_{s}{\hat{a}}_{ijs}\;=\sum_{s=1}^{m}\;\frac{\omega_{s}}{n}\sum_{l=1}^{n}\;(a_{ils}+a_{ljs}-0.5)\;=\sum_{s=1}^{m}\;\frac{1}{n}\sum_{l=1}^{n}\;(\omega_{s}a_{ils}+\omega_{s}a_{ljs}-0.5\omega_{s})\;=\sum_{l=1}^{n}\;\sum_{s=1}^{m}\;\frac{1}{n}(\omega_{s}a_{ils}+\omega_{s}a_{ljs}-0.5\omega_{s})\;=\frac{1}{n}\sum_{l=1}^{n}\;(a_{il}+a_{lj}-0.5)$$

Thus, we have ${\hat{\bar{a}}}_{ij}={\hat{a}}_{ij}$, that is, $\hat{A}=\hat{\bar{A}}$. This leads to the following result.

**Theorem 8.**  *The optimal estimation matrix of weighted arithmetic average combination of individual FPRs is equivalent to the weighted arithmetic average combination of the optimal estimation matrix of individual FPRs.*

### 5.2. Construction of Consensus Measure for Collective FPR

#### 5.2.1. The Case of Additively Consistent FPR

Let $A={\left(a_{ij}\right)}_{n\times n}$ be the weighted arithmetic average combination of the FPRs $A_{s}={\left(a_{ijs}\right)}_{n\times n}$, s=1,2,…,m. That is, $a_{ij}=\sum_{s=1}^{m}\;\omega_{s}a_{ijs}$, where $\omega_{s}$ satisfying $\sum_{s=1}^{m}\;\omega_{s}=1$ represents the relative importance of DM $d_{s}$, s∈M. Let $\hat{A}={\left({\hat{a}}_{ij}\right)}_{n\times n}$ be the corresponding optimal estimation matrix of $A={\left(a_{ij}\right)}_{n\times n}$. 

According to Theorem 7, if $A={\left(a_{ij}\right)}_{n\times n}$ is an additively consistent FPR, then $\hat{A}={\left({\hat{a}}_{ij}\right)}_{n\times n}$ is also an additively consistent FPR. If $A={\left(a_{ij}\right)}_{n\times n}$ is not consistent, then there exists at least one pair i,j∈N, such that ${\hat{a}}_{ij}\neq a_{ij}$. The smaller distance between ${\hat{a}}_{ij}$ and $a_{ij}$ is, the better consensus of the estimation. This also denotes that, the bigger deviation between ${\hat{a}}_{ij}$ and $a_{ij}$ is, the higher non-consensus of the estimation. 

Let $\delta_{ij}=|{\hat{a}}_{ij}-a_{ij}|$ denote the deviation between ${\hat{a}}_{ij}$ and $a_{ij}$. We propose three consensus measures between the collective FPR and its optimal estimation matrix as follows. 

(I). Consensus degree on a pair of alternatives $x_{i}$ and $x_{j}$.

**Definition 4.**  *The deviation* $gc_{ij}=1-\delta_{ij}$ *is called the consensus degree on the pair of alternatives* $x_{i}$ *and* $x_{j}$*, and the matrix* $GCM={\left(gc_{ij}\right)}_{n\times n}$ *is called consensus degree associated with all the pairs of alternatives* $x_{i}$ *and* $x_{j}$*,* i,j∈N*.*

The smaller $\delta_{ij}$ (or the bigger $1-\delta_{ij}$) is, the higher consensus of the estimation associated with $a_{ij}$. 

(II). Consensus degree on an alternative $x_{i}$.

**Definition 5.**  $gc_{i}=\sum_{j=1,j\neq i}^{n}\;gc_{ij}/(n-1)$ *is called the consensus degree on the alternative* $x_{i}$ *, and* $GCV={\left(gc_{1}\;gc_{2}\;\ldots \;gc_{n}\right)}^{T}$ *the consistency degree vector associated with all the alternatives.*

The number $gc_{i}$ is actually an index for measuring the consensus degree between the optimal estimation and the original estimation of the alternative $x_{i}$. In other words, the bigger the value of $gc_{i}$ is, the higher consensus of the estimation associated with the alternative $x_{i}$, i∈N. 

(III). Consensus degree on the FPR $A={\left(a_{ij}\right)}_{n\times n}$.

**Definition 6.**  $GC=\sum_{i=1}^{n}\;gc_{i}/n$ *is the called consensus degree of the collective FPR* $A={\left(a_{ij}\right)}_{n\times n}$*.*

The bigger the value of GC is, the higher consensus on the FPR $A_{s}={\left(a_{ijs}\right)}_{n\times n}$, s,s∈M. The consensus measure is also similar to that of Chiclana et al. [31].

**Example 2.**  *The FPRs* $A_{s}$*,* s=1,2,3,4*, as presented by Chiclana* [31]*, are given as follows:*

$$A_{1}=\left(\begin{array}{cccc} 0.5 & 0.2 & 0.6 & 0.4\\ 0.8 & 0.5 & 0.9 & 0.7\\ 0.4 & 0.1 & 0.5 & 0.3\\ 0.6 & 0.3 & 0.7 & 0.5 \end{array}\right);A_{2}=\left(\begin{array}{cccc} 0.5 & 0.7 & 0.9 & 0.5\\ 0.3 & 0.5 & 0.6 & 0.7\\ 0.1 & 0.4 & 0.5 & 0.8\\ 0.5 & 0.3 & 0.2 & 0.5 \end{array}\right);\;A_{3}=\left(\begin{array}{cccc} 0.5 & 0.3 & 0.5 & 0.7\\ 0.7 & 0.5 & 0.1 & 0.3\\ 0.5 & 0.9 & 0.5 & 0.25\\ 0.3 & 0.7 & 0.75 & 0.5 \end{array}\right);A_{4}=\left(\begin{array}{cccc} 0.5 & 0.25 & 0.15 & 0.65\\ 0.75 & 0.5 & 0.6 & 0.8\\ 0.85 & 0.4 & 0.5 & 0.5\\ 0.35 & 0.2 & 0.5 & 0.5 \end{array}\right).$$

Suppose that the relative weight of each DM is 0.25. Then, the arithmetic average combination of the FPRs $A_{i},i=1,2,3,4$, is

$$A=\left(\begin{array}{cccc} 0.5000 & 0.3625 & 0.5375 & 0.5625\\ 0.6375 & 0.5000 & 0.5500 & 0.6250\\ 0.4625 & 0.4500 & 0.5000 & 0.4625\\ 0.4375 & 0.3750 & 0.5375 & 0.5000 \end{array}\right)$$

According to Section 3.2, the corresponding additively consistent FPR is

$$\hat{A}=\left(\begin{array}{cccc} 0.5000 & 0.4125 & 0.5219 & 0.5281\\ 0.5875 & 0.5000 & 0.6094 & 0.6156\\ 0.4781 & 0.3906 & 0.5000 & 0.5062\\ 0.4719 & 0.3844 & 0.4938 & 0.5000 \end{array}\right)$$

The consensus measures of the collective FPR A as proposed in this paper and by Chiclana, respectively, are listed in Table 2. 

Here, we have two different consensus measures. The first consensus measure (our measure) is based on the optimal estimation matrix. That is, we regard this matrix as an objective matrix. The consensus degree of the FPR is based on the differences between the collective FPR (aggregated by arithmetic average of individual FPRs) and its optimal estimation FPR. In this sense, the first consensus measure is constructed from the point of view of collective divergence. The second consensus (Chiclana’s measure [31]) is based on the differences among all the individual FPRs. In this sense, the second consensus measure is constructed from the point of view of individual divergence. Consequently, the numerical values of the first consensus measure are greater than that of the second consensus measure, as shown in Table 2. 

#### 5.2.2. The Case of Multiplicative Consistent FPRs

Let $A={\left(a_{ij}\right)}_{n\times n}$ be the weighted geometric average combination of the FPRs $A_{s}={\left(a_{ijs}\right)}_{n\times n}$, s=1,2,…,m. That is, $a_{ij}=\frac{\prod_{s=1}^{m}\;{\left(a_{ijs}\right)}^{\omega_{s}}}{\prod_{s=1}^{m}\;{\left(a_{ijs}\right)}^{\omega_{s}}+\prod_{s=1}^{m}\;{\left(a_{jis}\right)}^{\omega_{s}}}$, where $\omega_{s}$ satisfying $\sum_{s=1}^{m}\;\omega_{s}=1$ stands for the relative importance of DM $d_{s}$, s∈M. Let $\hat{A}={\left({\hat{a}}_{ij}\right)}_{n\times n}$ be the corresponding optimal estimation matrix of $A={\left(a_{ij}\right)}_{n\times n}$. Let $\delta_{ij}=|{\hat{a}}_{ij}-a_{ij}|$ denote the distance between ${\hat{a}}_{ij}$ and $a_{ij}$. Similar to Section 5.2.1, three consensus measures between the collective FPR and its optimal estimation matrix from the multiplicative consistency point of view are proposed as follows. 

(I). Consensus degree on a pair of alternatives $x_{i}$ and $x_{j}$.

**Definition 7.**  $gc_{ij}=1-\delta_{ij}$ *is called the consensus degree on the pair of alternatives* $x_{i}$ *and* $x_{j}$*, and the matrix* $GCM={\left(gc_{ij}\right)}_{n\times n}$ *the consensus degree associated with all pairs of alternatives* $x_{i}$ *and* $x_{j}$*,* i,j∈N*.*

(II). Consensus degree on an alternative $x_{i}$.

**Definition 8.**  $gc_{i}=\sum_{j=1,j\neq i}^{n}\;gc_{ij}/(n-1)$ *is called the consensus degree on the alternative* $x_{i}$*,* $GCV={\left(gc_{1}\;gc_{2}\;\ldots \;gc_{n}\right)}^{T}$ *the consensus degree vector associated with all the alternatives.*

(III). Consensus degree on the FPR $A={\left(a_{ij}\right)}_{n\times n}$.

**Definition 9.**  $GC=\sum_{i=1}^{n}\;gc_{i}/n$ *is called consensus degree of the collective FPR* $A={\left(a_{ij}\right)}_{n\times n}$*.*

#### 5.2.3. The Algorithm for Consensus Measure of the FPRs

Step 1. Construct the optimal estimation matrix of the FPRs by using Equation (8) or Equation (11). 

Step 2. Aggregate the individual FPRs into a collective FPR by using the weighted geometric average or the weighted arithmetic average method. 

Step 3. Compute the consensus degree matrix $GCM={\left(gc_{ij}\right)}_{n\times n}$, consistency degree vector $GCV={\left(gc_{1}\;gc_{2}\;\ldots \;gc_{n}\right)}^{T}$, and the consensus degree of all the FPRs $A_{s}={\left(a_{ijs}\right)}_{n\times n},s\in M$ by using Definitions 4–9. 

In the following, we use an example to show the process of measuring the consensus of the FPRs.

## 6. Case Study

Taking the ecological security evaluation of five important cities, Shanghai, Nanjing, Hangzhou, Suzhou and Wuxi, in the Yangtze River Delta as an example, with reference to the natural environment, ecological environment disaster, environmental pollution, social and economic indicators, experts $d_{1},d_{2},d_{3}$ made the comprehensive evaluation of ecological security of the five cities according to their own experience. Through pairwise comparison, the fuzzy preference relation $A_{1},A_{2},A_{3}$ is constructed to achieve the regional ecological grading of the five cities.

Due to the limited rationality of experts and the incompleteness of knowledge, in the process of making judgments, it is often difficult for experts to ensure logical consistency. Based on the consistency measure and the consensus measure method proposed in this paper, the consistency of the logic and the validity of the judgment matrix are examined in the expert judgment process considering the consistency and multiplicative consistency of the FPR.

Suppose that there are three FPRs $A_{1},A_{2},A_{3}$ presented by DMs $d_{1},d_{2},d_{3}$, and that the relative weights of the DMs are 0.2, 0.4, and 0.4, respectively, where

$$A_{1}=\left(\begin{array}{ccccc} 0.5 & 0.6 & 0.5 & 0.9 & 0.7\\ 0.4 & 0.5 & 0.7 & 0.3 & 0.5\\ 0.5 & 0.3 & 0.5 & 0.6 & 0.4\\ 0.1 & 0.7 & 0.4 & 0.5 & 0.3\\ 0.3 & 0.5 & 0.6 & 0.7 & 0.5 \end{array}\right);A_{2}=\left(\begin{array}{ccccc} 0.5 & 0.5 & 0.4 & 0.8 & 0.9\\ 0.5 & 0.5 & 0.8 & 0.2 & 0.4\\ 0.6 & 0.2 & 0.5 & 0.7 & 0.4\\ 0.2 & 0.8 & 0.3 & 0.5 & 0.4\\ 0.1 & 0.6 & 0.6 & 0.6 & 0.5 \end{array}\right);A_{3}=\left(\begin{array}{ccccc} 0.5 & 0.4 & 0.7 & 0.3 & 0.9\\ 0.6 & 0.5 & 0.6 & 0.5 & 0.8\\ 0.3 & 0.4 & 0.5 & 0.6 & 0.4\\ 0.7 & 0.5 & 0.4 & 0.5 & 0.5\\ 0.1 & 0.2 & 0.6 & 0.5 & 0.5 \end{array}\right).$$

Step 1. According to Equation (8), the optimal estimation matrices of $A_{1}$, $A_{2}$, $A_{3}$ are ${\hat{A}}_{1}$, ${\hat{A}}_{2}$, ${\hat{A}}_{3}$, respectively.

$${\hat{A}}_{1}=\left(\begin{array}{ccccc} 0.5000 & 0.6838 & 0.7025 & 0.7704 & 0.6477\\ 0.3162 & 0.5000 & 0.5221 & 0.6081 & 0.4595\\ 0.2975 & 0.4779 & 0.5000 & 0.5869 & 0.4377\\ 0.2296 & 0.3919 & 0.4131 & 0.5000 & 0.3540\\ 0.3523 & 0.5405 & 0.5623 & 0.6460 & 0.5000 \end{array}\right);\;\begin{array}{l} {\hat{A}}_{2}=\left(\begin{array}{ccccc} 0.5000 & 0.6719 & 0.6777 & 0.7081 & 0.6967\\ 0.3281 & 0.5000 & 0.5067 & 0.5423 & 0.5287\\ 0.3223 & 0.4933 & 0.5000 & 0.5356 & 0.5221\\ 0.2919 & 0.4577 & 0.4644 & 0.5000 & 0.4864\\ 0.3033 & 0.4713 & 0.4779 & 0.5136 & 0.5000 \end{array}\right);\\ {\hat{A}}_{3}=\left(\begin{array}{ccccc} 0.5000 & 0.4797 & 0.6477 & 0.5671 & 0.7299\\ 0.5203 & 0.5000 & 0.6660 & 0.5869 & 0.7456\\ 0.3523 & 0.3340 & 0.5000 & 0.4161 & 0.5951\\ 0.4329 & 0.4131 & 0.5839 & 0.5000 & 0.6735\\ 0.2701 & 0.2544 & 0.4049 & 0.3265 & 0.5000 \end{array}\right). \end{array}$$

For example, the (1,2) entry 0.6838 of ${\hat{A}}_{1}$ is obtained as follows:

$$\frac{{\left((0.5\times 0.6\times 0.5\times 0.9\times 0.7\times 0.6\times 0.5\times 0.3\times 0.7\times 0.5)/(0.5\times 0.4\times 0.5\times 0.1\times 0.3\times 0.4\times 0.5\times 0.7\times 0.3\times 0.5)\right)}^{0.2}}{1+{\left((0.5\times 0.6\times 0.5\times 0.9\times 0.7\times 0.6\times 0.5\times 0.3\times 0.7\times 0.5)/(0.5\times 0.4\times 0.5\times 0.1\times 0.3\times 0.4\times 0.5\times 0.7\times 0.3\times 0.5)\right)}^{0.2}}=0.6838.$$

Step 2. The weighted geometric average combination A of $A_{1},A_{2},A_{3}$ is

$$A=\left(\begin{array}{ccccc} 0.5000 & 0.4797 & 0.5441 & 0.6581 & 0.8729\\ 0.5203 & 0.5000 & 0.7081 & 0.3265 & 0.5968\\ 0.4559 & 0.2919 & 0.5000 & 0.6416 & 0.4000\\ 0.3419 & 0.6735 & 0.3584 & 0.5000 & 0.4178\\ 0.1271 & 0.4032 & 0.6000 & 0.5822 & 0.5000 \end{array}\right)$$

According to Equation (16), we also take the (1,2) entry 0.4797 of A as an example to illustrate how matrix A is obtained:

$$\frac{{\left(0.6/0.4\right)}^{0.2}\times {\left(0.5/0.5\right)}^{0.4}\times {\left(0.4/0.6\right)}^{0.4}}{1+{\left(0.6/0.4\right)}^{0.2}\times {\left(0.5/0.5\right)}^{0.4}\times {\left(0.4/0.6\right)}^{0.4}}=0.4797$$

Step 3. The weighted geometric average combination $\hat{A}$ of ${\hat{A}}_{1},{\hat{A}}_{2},{\hat{A}}_{3}$ is

$$\hat{A}=\left(\begin{array}{ccccc} 0.5000 & 0.6007 & 0.6710 & 0.6692 & 0.7010\\ 0.3993 & 0.5000 & 0.5755 & 0.5735 & 0.6091\\ 0.3290 & 0.4245 & 0.5000 & 0.4979 & 0.5348\\ 0.3308 & 0.4265 & 0.5021 & 0.5000 & 0.5368\\ 0.2990 & 0.3909 & 0.4652 & 0.4632 & 0.5000 \end{array}\right).$$

According to Equation (16), we take the (1,2) entry 0.6007 of $\hat{A}$ as an example:

$$\frac{{\left(0.6838/0.3162\right)}^{0.2}\times {\left(0.6719/0.3281\right)}^{0.4}\times {\left(0.4797/0.5203\right)}^{0.4}}{1+({\left(0.6838/0.3162\right)}^{0.2}\times {\left(0.6719/0.3281\right)}^{0.4}\times {\left(0.4797/0.5203\right)}^{0.4}}=0.6007$$

In fact, $\hat{A}$ can also be obtained from A using Equation (8). 

Step 4. The consensus degree matrix $GC={\left(gc_{ij}\right)}_{n\times n}$ is given below, where $gc_{ij}=1-\delta_{ij}$, and $\delta_{ij}=|{\hat{a}}_{ij}-a_{ij}|$ i,j∈N

$$GC=\left(\begin{array}{ccccc} 1 & 0.8790 & 0.8731 & 0.9889 & 0.8281\\ 0.8790 & 1 & 0.8674 & 0.7530 & 0.9877\\ 0.8731 & 0.8674 & 1 & 0.8563 & 0.8652\\ 0.9889 & 0.7530 & 0.8563 & 1 & 0.8810\\ 0.8281 & 0.9877 & 0.8652 & 0.8810 & 1 \end{array}\right).$$

In particular, we look at how the (2,4) entry 0.7530 of GC is produced as an example, 1−|0.5735−0.3265|=0.7530. 

Step 5. The consensus vector $GV={\left(gc_{1}gc_{2}\ldots gc_{5}\right)}^{T}$ on the alternatives $x_{i},i=1,2,3,4,5$, is $GV={\left(0.8923,0.8718,0.8655,0.8698,0.8905\right)}^{T}$. Taking 0.8923 as an example, it is obtained by (0.8790+0.8731+0.9889+0.8281)/4. 

Step 6. The global consensus of $A={\left(a_{ij}\right)}_{n\times n}$ is 0.8780, which is produced by (0.8923+0.8718+0.8655+0.8698+0.8905)/5. 

The above research shows that the degree of consistency of the three experts’ comprehensive evaluation of ecological security for five cities is 0.8780, which has a high degree of consensus. It also shows that the overall judgment of three experts have a certain degree of reliability.

## 7. Conclusions

The evaluation of regional ecological security risk by group decision-making method can not only make full use of the subjective experience of expert groups, but also overcomes the difficulties of complex objective structure and incomplete objective data to a certain extent. Based on the theory of consensus decision making of fuzzy preference relation, this paper studies the consistency test method of expert opinion in ecological security assessment. In this paper, we proposed our consistency measure model by computing the distance between the original individual FPR and its optimal estimation matrix, and consensus measure model by computing the distance between the original collective FPR and its optimal estimation matrix. The contribution of this work can be seen from several different angles.

(1) Every entry in an inconsistent FPR is useful for the reason that it reflects the overall thinking of a DM. In this paper, we use the method of optimization to obtain the optimal additively consistent or the optimal multiplicative consistent FPR. The optimization method is in fact either the geometric average or the arithmetic average. It comprehensively considers each entry in the inconsistent FPR. Consequently, all information in the inconsistent FPR is fully used.
(2) Every FPR in GDM is useful for the reason that it reflects the influence of each individual in the group. In this paper, we use the weighted geometric average or the weighted arithmetic average to aggregate individual FPRs into a collective FPR. We also show by two theorems that the optimal estimation matrix of the aggregated FPR is equivalent to the aggregated optimal estimation matrix of different individual FPRs. The theorems indicate additionally that there is no information loss that occurs during the aggregation of the individual FPRs and reconstruction of the optimal collective FPR.
(3) The optimal FPRs are helpful in measuring the consistency degree of individual judgment and the consensus degree of collective judgment. Being similar to the consensus model as proposed by Chiclana [31], a new consistency measure and new consensus measure, established on the distance between the original estimation and the optimal estimation, are developed. We also show by using two examples initially constructed by Chiclana et al. that the new models can better reflect the consistency degree of individual judgments and the consensus degree of collective judgments.
(4) Additionally, we want to mention that our consensus measure is based on the deviation between the original collective estimation and its optimal estimation, while Chiclana’s consensus measure [31] on the deviation among all the individual estimation. In [31], Chiclana et al. also suggested a consensus threshold, named the minimum level of agreement by the group of DMs. Our next paper will cover this issue by using simulation on random data.

## Figures and Tables

**Table 1:** The Consistency Measures of the Preference Relation.

	Pairs of Alternatives (CM)	Alternatives (CV)	Relation (C)
Our method	$\left(\begin{array}{cccc} 1.0000 & 0.9250 & 0.8000 & 0.7250\\ 0.9250 & 1.0000 & 0.9750 & 0.9500\\ 0.8000 & 0.9750 & 1.0000 & 0.7750\\ 0.7250 & 0.9500 & 0.7750 & 1.0000 \end{array}\right)$	${\left(\begin{array}{c} 0.8167\\ 0.9500\\ 0.8500\\ 0.8167 \end{array}\right)}^{T}$	0.8583
Chiclana’s method	$\left(\begin{array}{cccc} 1.0000 & 0.8500 & 0.6000 & 0.5000\\ 0.8500 & 1.0000 & 0.9500 & 0.9000\\ 0.6000 & 0.9500 & 1.0000 & 0.5500\\ 0.5000 & 0.9000 & 0.5500 & 1.0000 \end{array}\right)$	${\left(\begin{array}{c} 0.6500\\ 0.9000\\ 0.7000\\ 0.6500 \end{array}\right)}^{T}$	0.7300

**Table 2:** The Consensus Measures of the Collective FPR.

	Pairs of Alternatives (GCM)	Alternatives (GCV)	Relation (GC)
Our method	$\left(\begin{array}{cccc} 1 & 0.95 & 0.9844 & 0.9656\\ 0.95 & 1 & 0.9406 & 0.9906\\ 0.9844 & 0.9406 & 1 & 0.9563\\ 0.9656 & 0.9906 & 0.9023 & 1 \end{array}\right)$	${\left(\begin{array}{c} 0.9667\\ 0.9604\\ 0.9604\\ 0.9528 \end{array}\right)}^{T}$	0.9601
Chiclana’s method	$\left(\begin{array}{cccc} 1 & 0.6700 & 0.7000 & 0.7400\\ 0.6700 & 1 & 0.7600 & 0.7500\\ 0.7000 & 0.7600 & 1 & 0.8700\\ 0.7400 & 0.7500 & 0.8700 & 1 \end{array}\right)$	${\left(\begin{array}{c} 0.7100\\ 0.7300\\ 0.7800\\ 0.7900 \end{array}\right)}^{T}$	0.7500
